# Stress–Strain Properties and Gas Permeability Evolution of Hybrid Fiber Engineered Cementitious Composites in the Process of Compression

**DOI:** 10.3390/ma12091382

**Published:** 2019-04-28

**Authors:** Zhenbo Wang, Jianping Zuo, Chang Liu, Zishan Zhang, Yudong Han

**Affiliations:** 1 School of Mechanics and Civil Engineering, China University of Mining and Technology, Beijing 100083, China; zqt1700601012g@student.cumtb.edu.cn (C.L.); sqt1700601022@student.cumtb.edu.cn (Z.Z.); 2 Central Research Institute of Building and Construction, MCC Group Co., Ltd., Beijing 100088, China; hanyudong@cribc.com

**Keywords:** engineered cementitious composites, hybrid fiber, triaxial compression, permeability

## Abstract

Polyvinyl alcohol (PVA)-steel hybrid fiber reinforced engineered cementitious composites (ECC) characterized by optimal combination of high strength and high ductility were developed recently. These composites exhibit even tighter crack width than normal ECC, showing great potential for lower permeability in cracked state, and consequently improving the durability of ECC structures. In addition, the wide variety of promising applications in underground or hydraulic structures calls for knowledge on the mechanical behavior and corresponding permeability properties of strained ECC under multiaxial stress, as they are essential for structural analysis and durability design. Experimental investigations into the compressive properties and the in-situ gas permeability of PVA-steel hybrid fiber ECC were performed in this study, with special focus on the impact of additional steel fiber content and confining pressure. The test results show that the presence of a low confinement level allows ECC to attain a substantial improvement on compressive behavior but impairs the enhancement efficiency of additional steel fiber. The permeability evolution of strained ECC corresponds to the variation of radial strains, both of which experience little change below the threshold stress but a rapid increase beyond the peak axial strain. Apart from exhibiting low permeability at relatively small strains in the pre-peak stage, ECC can also exhibit low permeability at higher levels of compressive strain up to 2.0%. However, unlike the case in tensile loading, impermeability of cracked ECC in compression would be weakened by additional steel fibers, especially in the post-peak stage. The present research is expected to provide insight into performance-based durability design of structures made of or strengthened with ECC.

## 1. Introduction

In concrete construction, cracks are known to be one of the most serious issues concerning the decrease of durability. Under structural and/or environmental loads, cracks will open quickly to a macroscopically visible level owing to the low tensile strength and strain-softening properties of concrete. The presence of widely opened cracks accelerates the deterioration and the consequent corrosion process of reinforced concrete by constituting preferential paths for deleterious matter ingress. While extensive work has been done with regard to uncracked concrete [1,2,3], the transport properties of cracked concrete appear to play a more decisive role in the long-term durability. It has been recognized that the permeability of concrete increases rapidly with the growth of crack width [4,5]. As such, crack control in concrete becomes a critical concern for the development of sustainable concrete infrastructures.

Engineered Cementitious Composites (ECC) is a particular class of high-performance fiber reinforced cementitious composite, which characterizes strain-hardening, quasi-ductile behavior accompanied by crack width and spacing control function up to the ultimate tensile strain [6,7]. The tensile ductility of ECC, resulted from the formation of numerous steady-state fine cracks, is generally two orders of magnitude higher than that of conventional concrete. Of special interest is the crack width of each individual crack designed to first widen steadily to approximately 100 μm and then maintain until peak stress [6]. The tight crack width is expected to have little effect on the permeability of ECC even under cracked state, thus contributing to extended service life [8]. It should be pointed out here that, the purpose of fracture toughness control and fiber dispersion allows ECC to incorporate only a small amount of aggregates, which results in large drying shrinkage during setting and hardening process. Restrained drying shrinkage will cause cracking of structures at early age, raising problems of insufficient durability. Fortunately, ECC with characteristics of low drying shrinkage (LSECC) has been developed recently and applied successfully in concrete pavement [9,10]. Hereby, low shrinkage characteristic gives ECC superior cracking resistance beyond conventional concrete; even if cracking occurs unavoidably as a result of the material’s response to external load and environment, tight crack width lends ECC low permeability properties. Thus, a highly durable cementitious composites can be expected to be obtained.

For quite some time now, significant studies have been conducted on the mechanical properties of mono-fiber reinforced ECC, such as polyvinyl alcohol (PVA) fiber or polyethylene (PE) fiber reinforced systems [6,7,8,9,10,11,12]. Based on these studies, it has been recognized that fiber reinforced cementitious composites with high modulus fiber possesses high strength but low strain capacity, such as steel and carbon fibers, whereas those containing relatively low modulus fibers, such as PVA and PE fibers, behave in the opposite manner [12,13,14]. To obtain an optimal balance between high strain capacity and high strength, blending different types of fibers into ECC matrix turn out to be an effective solution [13,14,15,16]. In previous research, the tensile, flexural and compressive performance of PVA-steel hybrid fiber reinforced engineered cementitious composites had been investigated comprehensively by the authors [17,18,19]. In those studies, satisfactory balance between high strength and high ductility was achieved for ECC. More importantly, the average crack width corresponding to the tensile strength was restricted to as narrow as 30 μm, even smaller than that in mono-fiber ECC. It is to be expected to design ECC materials with lower permeability and reinforced ECC construction with more durable serviceability, by using hybrid-fiber method which produces extremely tight crack width even under large strain.

The water permeability evolution of cracked ECC after tension has been investigated experimentally by Lepech and Li [20]. It could be shown that the unloaded permeability of ECC maintained low even under a tensile strain of 3.0%. Compared to reinforced mortar specimens with wider cracks, ECC exhibited a up to six orders of magnitude lower permeability. In considering the larger crack width of ECC under tension than that in residual state, Liu et al. [21] conducted an experimental study on the permeability behavior of ECC under tension, and more accurate and realistic permeability data were obtained. In these studies, it was observed that self-healing of microcracks imposed a salient influence on the permeability of ECC, which assisted in reducing the permeability coefficient over time. Since water–cementitious matrix interaction can lead to rehydration of previously unhydrated components, it is generally agreed that permeability to chemically unreactive media like glycol or nitrogen yields more realistic values than permeability to water, serving as representative of intrinsic transport properties induced solely by mechanical cracking [22,23]. In addition to tension, the understanding of the compressive behavior and corresponding permeability evolution of ECC is also crucial for the durability design and analysis of ECC structures [24]. Unfortunately, there still exists little information on this topic. Moreover, with the wide variety of potential applications of ECC in underground or hydraulic structures like tunnel lining, grouting reinforcement, containment vessels and dam, its behaviors under multiaxial stress states have become an important issue [25,26,27]. Hence, it is of significance to characterize the permeability properties of ECC under a simultaneous application of multiaxial stress, so as to correlate the intrinsic permeability with real stress state.

In this work, the stress–strain properties and gas permeability evolution of PVA-steel hybrid fiber reinforced engineered cementitious composites was fully investigated under both uniaxial and triaxial compression. Special focus is centered on the impact of additional steel fiber content and confining pressure. As such, two additional steel fiber contents were selected while the PVA fiber content remains constant in the experimental program. At first, the influence of additional steel fiber content on the uniaxial compressive performance of ECC was evaluated in terms of stress–strain relation, strength, elastic modulus, strain corresponding to peak stress and Poisson’s ratio. After that, the compressive stress–strain behavior of ECC under a low confinement level was presented, and a comparison of uniaxial and triaxial compression was made. Finally, the permeability evolution of ECC with both mono-fiber and hybrid-fiber reinforcement in the process of triaxial compression was presented, and a discussion was conducted on the correlation between the permeability properties and cracking patterns of the materials. The research findings are expected to contribute to performance-based durability design of structures made of or strengthened with ECC.

## 2. Experimental Program

### 2.1. Materials and Specimen Preparation

The cement used in the mix was a newly developed composite cement with characteristic of low drying shrinkage [9], and its chemical content was detailed elsewhere [17]. The aggregate used was a fine silica sand with a maximum grain size of 150 μm and a mean size of 100 μm. In all mixtures, the water-cement ratio (w/c) and sand-cement ratio (s/c) was controlled at 0.25 and 0.20, respectively. A commercially available polycarboxylate superplasticizer was added at a dosage of 1.0% by mass of cement to achieve better workability and fiber distribution. The PVA fibers and micro steel fibers were manufactured by Kuarary Company (Tokyo, Japan) and Changhong Company (Anshan, China), respectively. Table 1 lists the dimensional information and mechanical properties of the fibers. In mix design, the volume fraction of PVA fibers was kept at a moderate volume fraction of 1.7%, which was commonly adopted in ECC mixtures, while the volume fractions of steel fibers varied at 0.0%, 0.6%, and 1.0%. Thus, three test series were formed as summarized in Table 2, which were designated as Mono, Hy1 and Hy2 respectively, according to the content of additional steel fibers. In addition, the apparent density *ρ* and permeability *k* of each mixture were measured, and the results were listed in Table 2 as well. The test method of permeability will be described in the next section.

When mixing ECC, the cement and fine silica sand were first dry-mixed for about 1 min using a mortar mixer at low speed. Later, water pre-mixed with superplasticizer was slowly added into the mixer. Wet mixing was continued for another 3–5 min until the paste reached a uniform state. The fibers were then gradually dispersed into the slurries with further 3 min to achieve uniform distribution. After that, the fresh mixture was poured into greased PVC molds in two equal layers with short-time vibrations. Then the surface of the fresh paste was carefully scraped for several times to achieve a favorable smoothness. After finishing the surface, the specimens were covered with plastic sheets for 24 h to prevent the possible damages induced by moisture loss. Immediately after demolding, the cylinders were moved into a standard curing room with the temperature and relative humidity controlled at 20 ± 2 °C and >95%, respectively. For each different mixture, a set of five cylinders with a diameter of 50 mm and a height of 100 mm were prepared. Among them, three specimens were used for uniaxial compression testing, and two specimens were used for triaxial compression and permeability testing. All cylinders were taken out at 28 days and carefully ground at both ends to guarantee parallel loading. In triaxial compression test, penetration of the pressure fluid into pores and microcracks on the surface of ECC was prevented by placing the specimen into heat-shrink tube. This tube can shrink and tightly wrap the side surface of the cylinder after a short-time heating. Besides applying isolation sleeve, efforts had been made to fill the visible pores on the specimen surface with hot melt adhesive to prevent sleeve damage in the presence of confining pressure in the chamber.

### 2.2. Test Methods

#### 2.2.1. Uniaxial Compression

**Figure 1:**
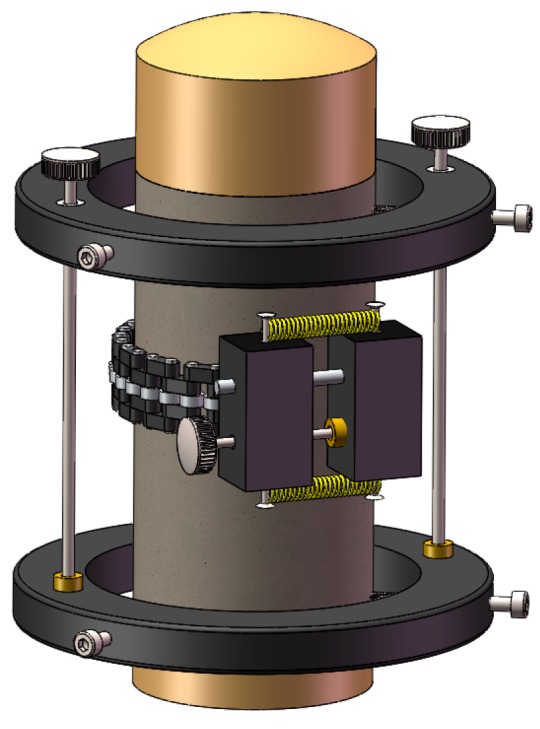
Experimental setup for uniaxial compression.

Firstly, uniaxial compression tests were carried out to obtain the complete stress–strain behaviors of PVA-steel hybrid fiber reinforced cementitious composites. The test was conducted using a servo-hydraulic testing machine with a 1000 kN capacity. Detailed experimental setup was displayed in Figure 1. Two axial extensometers and one circumferential extensometer were mounted at the central region of the specimen to measure the axial strain and the lateral strain, respectively. The actuator strain rate used for controlling the test was set to be 2 × 10^−4^ /min to obtain a stable softening stage of the stress–strain relations. During the loading process, the axial stress *σ*_1_, axial strain *ε*_1_ and radial strain *ε*_r_ were recorded simultaneously using a data acquisition system connected to the control system.

#### 2.2.2. Triaxial Compression

The triaxial compression tests under a confining pressure *σ*_3_ of 2.0 MPa were conducted to characterize the triaxial stress–strain properties of hybrid fiber reinforced composites using the servo-hydraulic testing machine. And in-situ permeability measurements were performed simultaneously in the compression process. Before applying the confining pressure, a small axial stress was imposed. Then, load control mode was employed to achieve the target confining pressure. After that, the test system was set to displacement control mode with a strain rate of 2 × 10^−4^/min, which is identical to that in uniaxial compression test. To allow measurement of in-situ permeability, the axial strain was increased by grading and each grading strain loaded 1000 μm/m in pre-peak stage or 2000 μm/m in post-peak stage, and the strain rate was slowed down to 1 × 10^−5^/min when each target grading strain was achieved.

#### 2.2.3. In Situ Permeability Test

**Figure 2:**
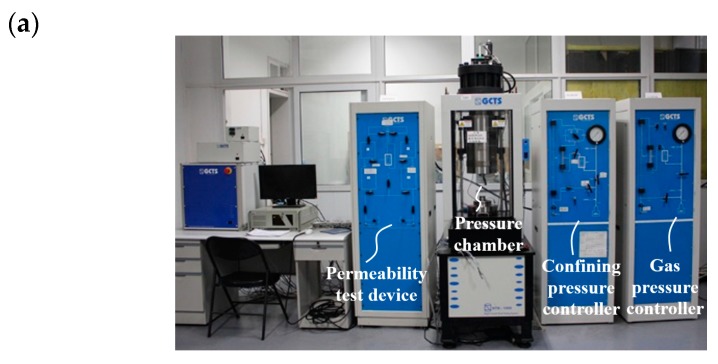

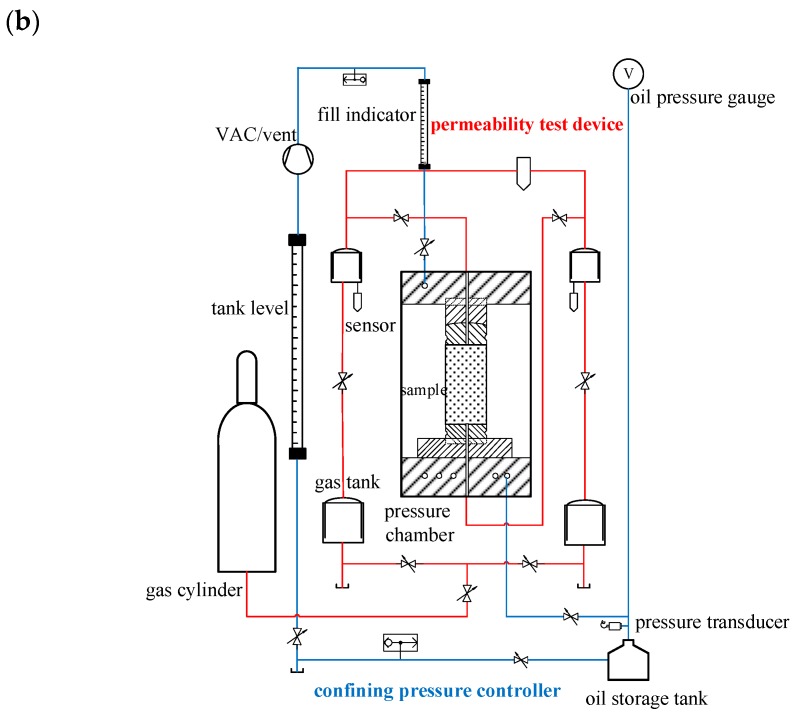
(**a**) Test system and (**b**) schematic diagram of permeability measure in the process of triaxial test.

In considering that water ingress in cementitious materials can result in rehydration of un-hydrated cement particles, thus altering the intrinsic transport properties induced by mechanical cracking, the permeability was measured using nitrogen gas as the neutral percolating medium. Under each strain level, pressure pulse method was adopted to rapidly determine the corresponding intrinsic permeability. The permeability measurement setup was illustrated in Figure 2. Firstly, certain pore pressure was imposed on the entire specimen until gas saturation, and then the pore pressure at the lower end was suddenly reduced, yielding a pore pressure pulse. Thus, a pressure difference was generated between the upper and lower ends, resulting in transient gas flow inside the specimen. The pore pressure difference was continuously reduced as nitrogen seeped in the sample, and the decay in a certain period of time could be recorded. Then, the permeability under certain stress state can be computed from the following formula

(1) $$k=\frac{\mu \beta VL}{2A\Delta t}\ln\left(\frac{\Delta P_{i}}{\Delta P_{f}}\right)$$

where, *k* is the permeability (m^2^), *μ* is the dynamic viscosity of the permeating fluid (Pa ∙ s), which is 1.78 × 10^−5^ Pa ∙ s for nitrogen, *β* is the volumetric compressibility factor of the permeating fluid (Pa^−1^), *V* is the volume of pressure vessel (m^3^), Δ*P*_i_ and Δ*P*_f_ are the respective initial and final pore pressure difference (Pa), *A* and *L* are the respective cross-sectional area (m^2^) and height (m) of the specimen, Δ*t* is the testing time (s). As is seen, it is not necessary to acquire the steady state of fluid seepage in pressure pulse method, which greatly shortens the testing time. By this method, the triaxial compression test on an individual specimen could be completed within 8 h, accompanied by the measurements of permeability at 14 strain levels.

## 3. Results and Discussion

### 3.1. Influence of Steel Fiber Content on Uniaxial Compressive Behavior

**Figure 3:**
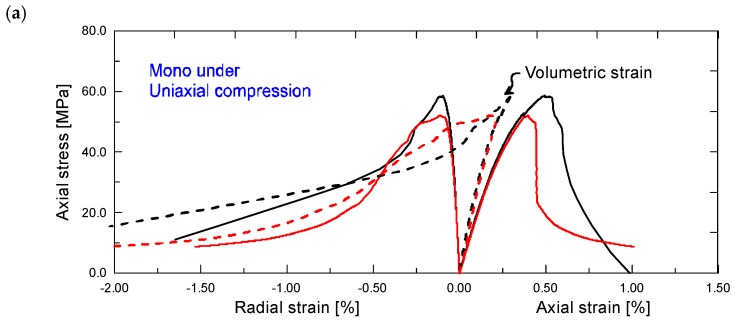

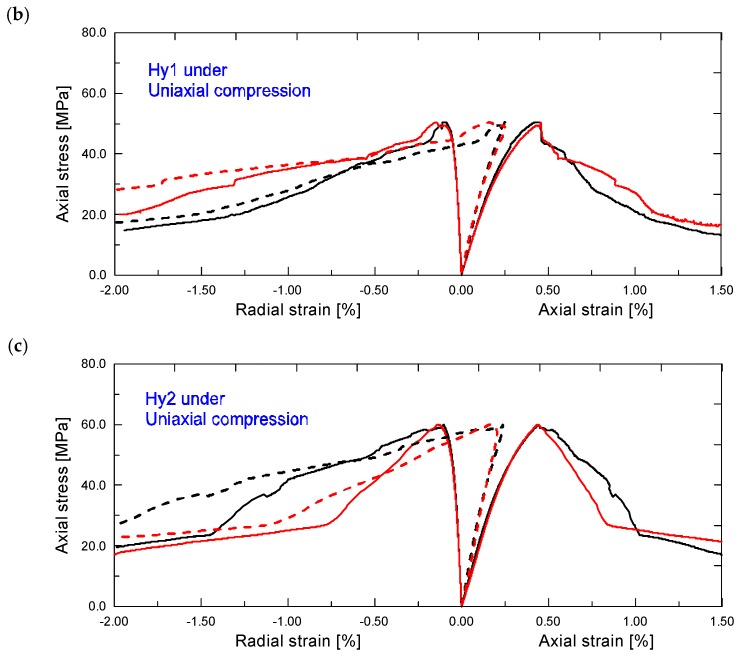
Stress–strain relations of (**a**) Mono, (**b**) Hy1 and (**c**) Hy2 mixtures under uniaxial compression.

Figure 3 shows the uniaxial compressive stress *σ*_1_-strain relations of mono and hybrid fiber reinforced cementitious composites at the age of 28 days, respectively. The axial strain *ε*_1_ is plotted in the positive direction, while the radial strain *ε*_r_ is plotted in the negative direction. And the stress *σ*_1_-volumetric strain *ε*_v_ curves of different ECC mixtures are provided as well in dashed line, where *ε*_v_ = *ε*_1_ + 2*ε*_r_.

**Figure 4:**
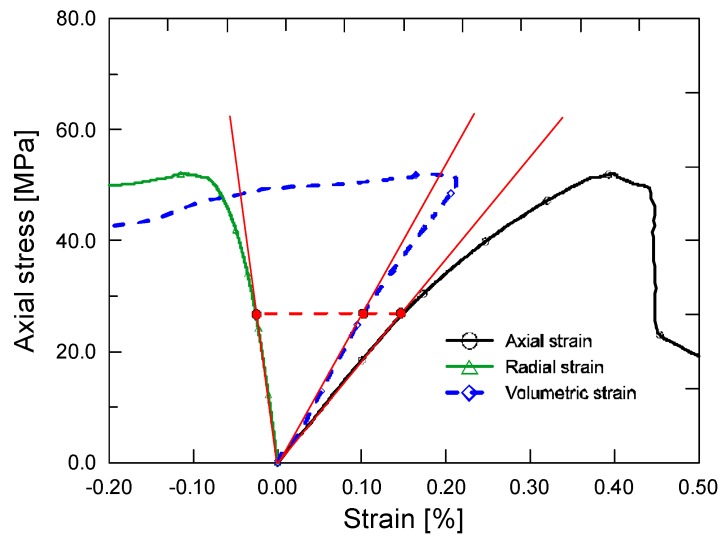
Illustration of the initial linear segments of stress–strain curves.

As figures show, all of the stress–strain curves can be divided into two stages: an ascending stage and a subsequent descending stage. In the ascending stage, the stress–strain curves behave first linear-elastically and then nonlinearly up to the peak stress *f*_c_. Note that the axial strain *ε*_1_, radial strain *ε*_r_ and volumetric strain *ε*_v_ all arrive at the end of the initial linear segment simultaneously, after which the curves begin to deviate appreciably from a straight line. This is explained graphically in Figure 4. The linear-elastic characteristic of ECC serving as a material property independent of the strain direction, is responsible for this result. When it comes to the descending stage, certain stress drop exists for mono PVA fiber reinforced mixture, followed by a subsequent residual stress segment which provides some toughness. The post-peak behavior in uniaxial compression appears not to be in accord with the characteristic tensile ductility of ECCs [6,7,8,9]. However, the compressive performance of ECC mixtures is significantly improved by incorporation of additional steel fibers. As the steel fiber content increases, the descending stage of the stress–strain curves tends to level off, thus resulting in higher residual stress. For example, when axial strain reaches 1.5%, the stress level of hybrid fiber mixtures with steel fiber content of both 0.6% and 1.0% still remains beyond 30% of the strength. That is, PVA-steel hybrid fiber reinforced cementitious composites maintains high load carrying capacity even under large compressive deformation, which is expected to be used to advantage in improving structural earthquake resistance.

**Figure 5:**
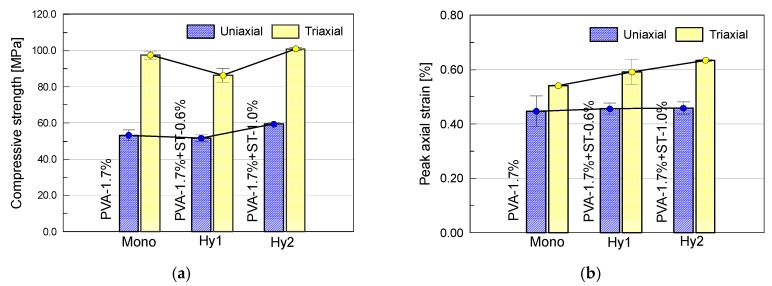

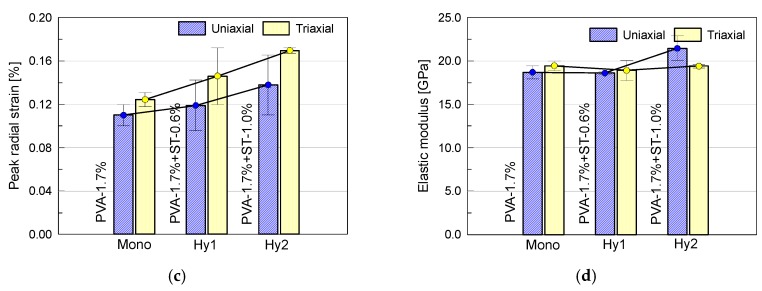
Effect of additional steel fiber content on (**a**) compressive strength, (**b**) peak axial strain, (**c**) peak radial strain and (**d**) elastic modulus of ECC under both uniaxial and triaxial compression.

The mechanical parameters determined by the stress–strain curves are summarized in Table 3, in which each data is attained by averaging three test results. In this table, *f*_c_ is the compressive strength, *E*_0_ is the elastic modulus, *ε*_0_ is the peak axial strain corresponding to the compressive strength, *ε*_02_ is the peak radial strain corresponding to the compressive strength and *ν*_0_ is the Poisson’s ratio. Figure 5 illustrates the influence of steel fiber content on each mechanical parameter of different ECC mixtures with and without confining pressure. Herein, each histogram bar presents an average value from two or three specimens. In this section, the effect of steel fiber on uniaxial compressive specimens is to be analyzed, while the triaxial specimens will be discussed in the next section.

The impact of steel fiber content on uniaxial compressive strength is found to be obvious. In general, increasing the volume fraction of steel fibers contributes to an increase tendency in strength. However, steel fiber content of 0.6% results in a slight decrease of compressive strength from 53.3 MPa to 51.7 MPa. As shown by the data in Table 2, the average apparent density of the Hy1 mixture is only 3 kg/m^3^ greater than that of the Mono mixture, which is not coincided with the fact that steel fiber content of 0.6% ought to allow a maximum of approximately 47 kg/m^3^ increase in weight. This might be partially attributed to the increase of porosity due to incorporation of steel fiber, which counteracts the enhancement effect of steel fiber. Thus, addition of steel fiber with volume fraction of 0.6% is accompanied by a slight degradation in strength instead. As the volume fraction of steel fiber increases further to 1.0%, the uniaxial compressive strength attains up to 59.4 MPa. It seems that the adverse effect of steel fiber addition tends to be reduced and eventually translated into a beneficial effect. This can be somewhat confirmed by the increased density of the Hy2 mixture.

The peak axial strains of each mixtures are increased slightly from 0.447% to 0.459% with the increase of steel fiber content from 0.0% up to 1.0%. By comparison, a relatively obvious increase from 0.110% to 0.138% occurs in peak radial strains. It means that the addition of steel fiber can improve the strain capacity, especially the lateral strain capacity of ECC under uniaxial compression. Due to the high stiffness and strength of steel fibers, the bridging stress across/along the cracks would be greatly strengthened, delaying the arrival of peak stress. Regarding the elastic modulus, a similar trend to that of compressive strength is observed. Exclusion of coarse aggregate from ECC matrix results in smaller elastic modulus within the range of 18.6–21.5 GPa, as compared to that of conventional concrete. In addition, there appears to be no consistent relationship between Poisson’s ratio and steel fiber content.

### 3.2. Stress–Strain Behaviors under Triaxial Compression

**Figure 6:**
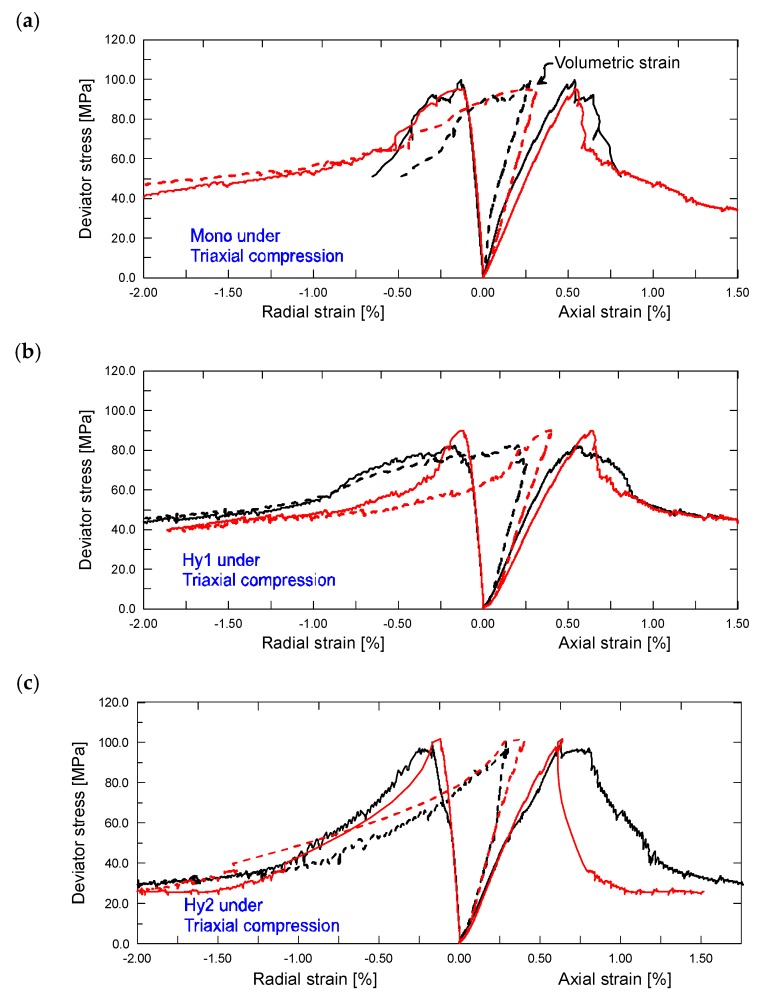
Stress–strain relations of (**a**) Mono, (**b**) Hy1 and (**c**) Hy2 mixtures under triaxial compression.

Figure 6 shows the triaxial compressive stress–strain relations of different ECC mixtures at the age of 28 days, respectively, where the vertical axis represents the differential stress, *σ*_1_-*σ*_3_. It can be seen that the resulting stress–strain curves are not as smooth as the case in uniaxial compression, since several in-situ permeability measurements were conducted at strain intervals of 1000 με or 2000 με.

It is observed that the stress–strain characteristic of ECC gains an important improvement under a confining pressure only of 2.0 MPa. With mono PVA fiber reinforced composites, stress drop existing in the descending stage of uniaxial compression tends to be eliminated, replaced by gradual stress degradation and higher residue stress. The confining pressure exerted on side surface of the specimen provides a uniform restriction, thus resulting in a considerable improvement on stress–strain behavior. As for ascending stage, the nonlinear segment following the liner-elastic segment exhibits decreased curvature in comparison to that in uniaxial compression. This speaks to the enhancement effect of confining pressure on the stiffness of specimen. Regarding the descending stage, all of the mixtures display typical toughness characteristic as observed in uniaxial compression of hybrid fiber composites.

**Figure 7:**
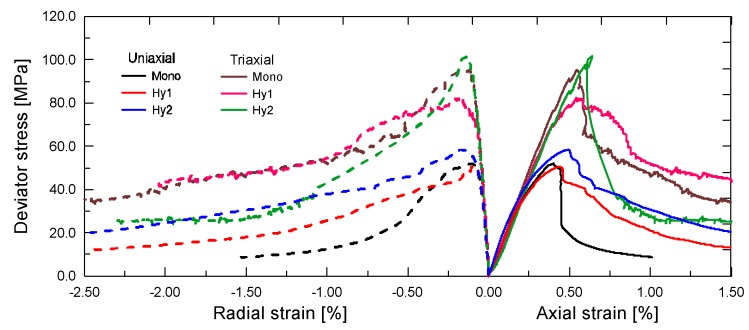
Comparison of typical stress–strain relations of engineered cementitious composites (ECC) mixtures with different steel fiber content under both uniaxial and triaxial compression.

To understand the effect of steel fiber on the stress–strain properties under uniaxial and triaxial compression, a comparison of typical compressive stress–strain relations of different ECC mixtures are presented in Figure 7, respectively. As is seen, all of the stress–strain curves can be divided into two distinct grades depending on their stress states. The confining pressure enables a substantial improvement on the compressive behavior of ECC. In particular, the magnitudes of both peak stress and residue stress seem to be doubled by the confining pressure. Under each stress state, addition of steel fibers still leads to a clear improvement on the stress–strain behavior. With the increase of steel fiber content, the ascending slope increases slightly, while the descending stage obtains a significant promotion, thus yielding higher residual stress. However, the positive responses of additional steel fiber appear to be somewhat impaired by the presence of confinement. This is understandable because confining pressure can provide a direct and stronger restraint on the lateral deformation, which will in turn limit the bridging efficiency of steel fiber. Therefore, the enhancement effect of steel fiber tends to be eliminated in the presence of confining pressure. In other words, steel fiber may be more suitable to be used in ECC components under uniaxial compression.

The determined mechanical parameters in triaxial compression are summarized in Table 3. The impact of steel fiber content on each mechanical parameter of ECC mixtures in the presence of confining pressure is illustrated in Figure 5. It shows that the strength under triaxial compression experiences a similar trend to that under uniaxial compression, although not to the same degree. Steel fiber content of 0.6% decreases the compressive strength from 97.5 MPa to 86.2 MPa, while steel fiber content of 1.0% attains a margin increment on compressive strength. The confinement appears to enlarge the adverse effect of steel fiber, i.e., inclusion of flaws and pores, but weaken its beneficial effect, i.e., enhancement on the fiber bridging effect across/along the cracks. It is of significance to point out that, with a confining pressure only of 2.0 MPa, the compressive strength achieves a significant increment of 35~45 MPa, which is much greater than that of normal or high-strength concrete reported in other literatures [24,25,26,27]. The compressive strength of ECC seems to be more sensitive to the exposed confining pressure. It might be because the confining pressure optimizes the bridging law across/along the cracks provided by PVA-steel hybrid fibers, thus yielding much more sufficient degree of cracking.

Both of the peak axial strains and radial strains are increased with the steel fiber content, which is similar to that in uniaxial compression. Moreover, it can be seen that the strain capacity of ECC is slightly improved by the confining pressure. To fracture ECC specimens subjected to the simultaneous action of both confining pressure and additional steel fibers, more strain energy is required. Regarding the elastic modulus, it shows little difference with the case in uniaxial compression, indicating little effect of low confinement on the stiffness of ECC.

**Figure 8:**
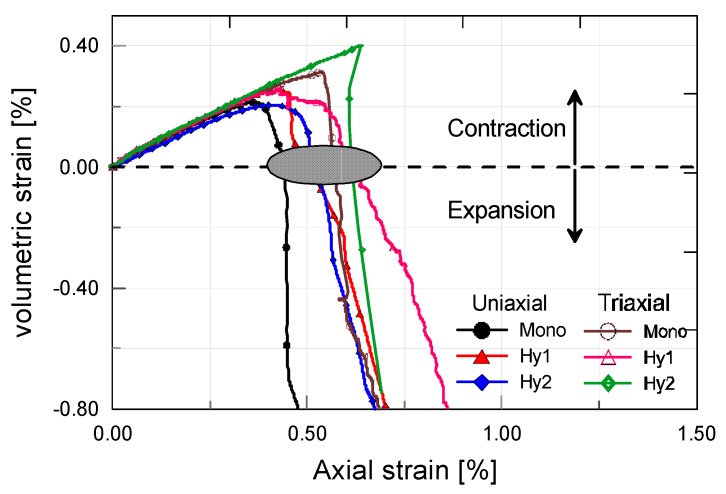
Volumetric strain-axial strain curves of different ECC mixtures under both axial and triaxial compression.

The variation of apparent volumetric strain along with the loading process is displayed in Figure 8, to advantage in understanding the compressive behavior of ECC. A horizontal dashed line is plotted to identify the contraction region and the expansion region. As is seen, the volumetric strain increases first linearly and then nonlinearly as the axial strain increases. It has been pointed out before that the linear-elastic characteristic of ECC is independent of the strain direction. This also holds true for the volumetric strain. After peak volumetric strain, all the curves encounter a sharp drop, giving an indication of rapid expansion in volume. Under triaxial compression of low confinement level or uniaxial compression, the strain energy release seems to be more violent than that of conventional concrete containing coarse aggregates [28]. However, with the propagation, opening and sliding of cracks, fiber bridging will take effect and contribute to the residue toughness, as shown in stress–strain curves. Figure 8 also highlights the points at which the volumetric strain intersects zero axis. These points follow a clear growing trend, where the axial strains at zero volumetric strain increase with the steel fiber content and the confining pressure. This demonstrates that the combined effect of additional steel fiber and confining pressure is clearly beneficial for improving the strain capacity of ECC in compression.

### 3.3. Permeability Evolution under Triaxial Compression (In Situ Measurements)

To investigate the evolution of intrinsic permeability characteristics of ECC mixtures, nitrogen is employed as the percolating medium to avoid the influence of water addition. Thus, a more realistic value reflecting the variation of internal cracking is believed to be obtained. Prior to loading, the initial permeability of different ECC mixtures were measured, and the results were listed in Table 2. It can be seen that, with the increase of steel fiber content, the permeability first increases and then decreases. The size and continuity of the pores and the interfacial transition zone would control the permeability in hardened ECC specimens. As discussed in Section 3.1, incorporation of steel fiber introduces more bubbles and flaws into the cementitious matrix and the fiber-matrix interface. Thus, larger permeability is resulted in hybrid fiber reinforced composites. Note that the hybrid fiber mixtures with steel fiber content of 1.0% attain a slightly smaller permeability than that with steel fiber content of 0.6%. This might be because a larger amount of steel fibers across the pores or flaws play a beneficial role in retarding fluid flow, which overcomes the adverse effect of increased pores due to steel fiber addition. In general, permeability and strength are closely related to each other through the porosity [28]. It is of interest to point out that, this may not always be the case for hybrid-fiber ECCs. For instance, addition of steel fibers into ECC mixture with volume fraction of 1.0% leads to a larger permeability but gives a higher strength. Although a number of pores and flaws are expected to be introduced, such a dosage of steel fibers can still provide sufficient enhancement that resist the spontaneous propagation and interlinkage of cracks, thus being responsible for the associated increase of strength.

**Figure 9:**
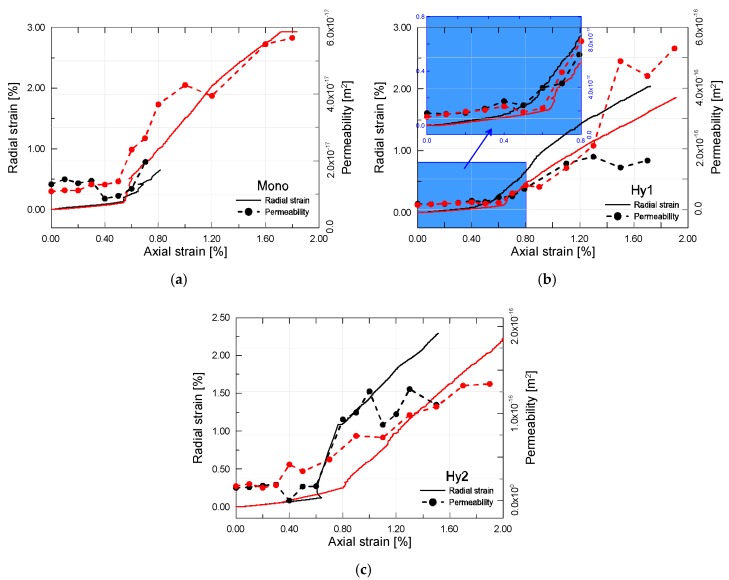
Permeability and radial strain evolution of (**a**) Mono, (**b**) Hy1 and (**c**) Hy2 in the process of compression test.

The measured permeability of cracked ECC mixtures as a function of axial strain is presented in Figure 9. As a reference, the variation of radial strains is depicted as well. It can be observed that the developing trend of different ECC mixtures are similar. The permeability of strained ECC increases first marginally and then rapidly, where the threshold value corresponds to the peak stress. Interestingly, the radial strain undergoes a similar developing trend. As the increment of lateral deformation increases during loading process, the permeability increases accordingly. The sudden change occurs almost simultaneously for both of the radial strain and the permeability. Thus, the variation of lateral strain can be reflective of the change in permeability during the loading process.

In the pre-peak stage, there exists a small change in the permeability at stress levels below the threshold values. Microcracks tend to be restricted to the fiber-matrix interface and the inherent voids at this stage, reflected by very little increase in permeability, if any. Compared to normal concrete, ECC mixtures all exhibit higher threshold stress values, ascribing it to the fiber bridging effect that delays the proliferation and propagation of microcracks in the matrix. Clearly, bridging the microcracks before they coalesce is beneficial since permeability is related to crack width. Beyond peak axial strain, the permeability encounters a rapid increase owing to the unstable opening, sliding and coalescence of cracks. In short, the crack system is becoming continuous due to the rapid propagation of cracks in both the matrix and the fiber-matrix interface. However, when the axial strain is larger than about 1.2%, the rate of increase of permeability becomes steady and less rapid. On must note that, in addition to exhibiting low permeability at relatively small strains in the pre-peak stage, ECC mixtures can also exhibit low permeability at higher levels of compressive strain up to 2.0% in the post-peak stage.

**Figure 10:**
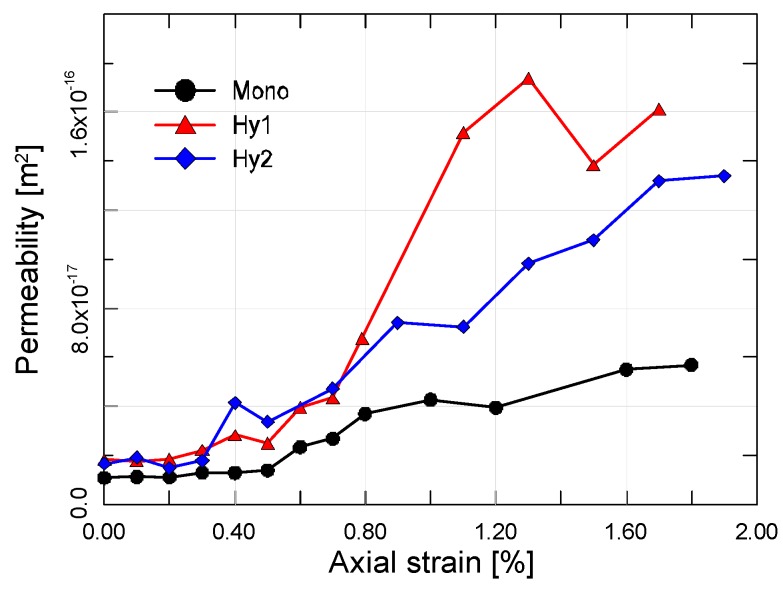
Comparison of typical permeability evolution curves of ECC mixtures with different steel fiber content.

As expected, incorporation of additional steel fibers plays a significant role in permeability evolution, cf. Figure 10. With the increase of axial strain, mono PVA fiber reinforced composites show a relatively moderate increment in permeability. It even stays unchanged at stress levels below the threshold values. By contrast, the hybrid fiber composites appear to be more permeable all along the loading process. It is interesting to point out here that the order of the initial permeability of all the mixtures, i.e., Hy1 > Hy2 > Mono, remains unchanged in both the pre-peak stage and the post-peak stage. Furthermore, the permeability differences inherited from the intact material turn out more and more pronounced with the increase of axial strain. From another point of view, the developing trend of permeability ought to be somewhat predicted by the initial permeability properties of each ECC mixture. The variations of permeability with increase of steel fiber content is likely due to the change in the crack profile [29]. It is to be expected that a multitude of closely spaced microcracks with poor connectivity form in mono PVA fiber composites, while a few larger cracks appear in hybrid fiber composites. Although the total crack width represented by radial strain is greater with the mono PVA fiber composites, the individual cracks are much finer than that in hybrid fiber composites, being responsible for the decreased permeability.

**Figure 11:**
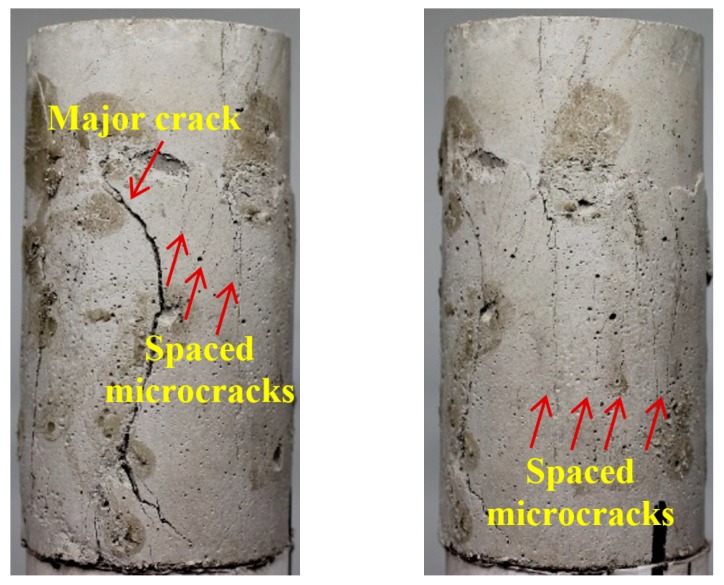
Crack pattern of Mono mixture after triaxial compression.

**Figure 12:**
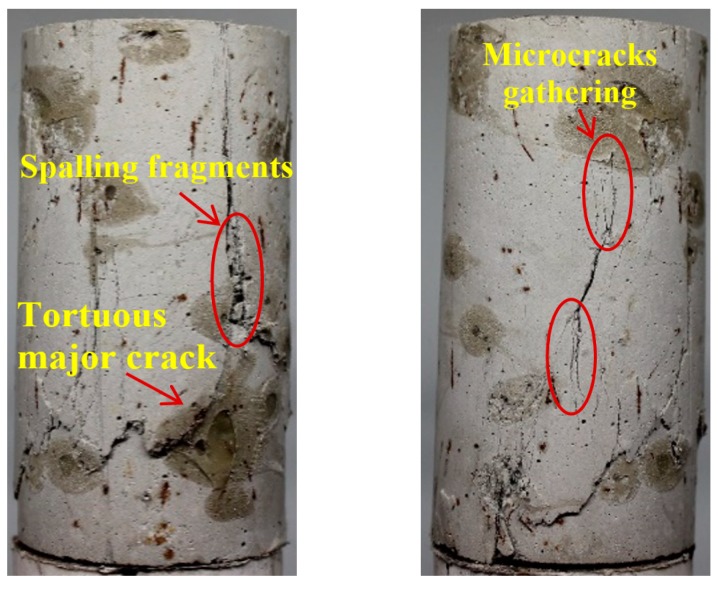
Crack pattern of Hy1 mixture after triaxial compression.

**Figure 13:**
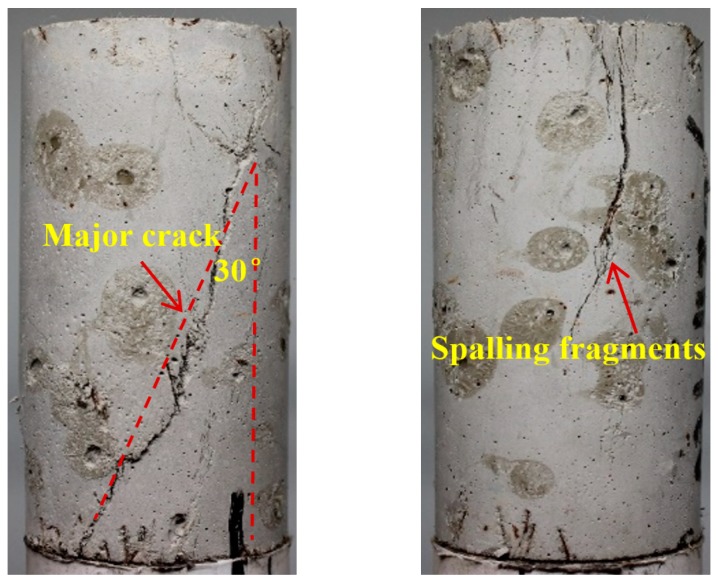
Crack pattern of Hy2 mixture after triaxial compression.

Typical failure patterns of different ECC mixtures after triaxial compression are shown in Figure 11, Figure 12 and Figure 13, where pictures taken from two different angles are displayed for each specimen. From Figure 11, the mono PVA fiber composites are found to be fractured with a tortuous main branch crack accompanied by closely spaced but disconnected microcracks. This phenomenon further confirms the preceding discussion on the permeability properties of mono PVA fiber composites. In contrast, the ECC specimens with hybrid fibers exhibit different failure modes. The addition of steel fibers results in more tortuous cracks inclined to the vertical plane, as shown in Figure 12 and Figure 13. And the higher strength and stiffness of steel fibers cause obvious damage on the cementitious matrixes, which form spalling fragments along the major cracks. Furthermore, it should be noticed that the microcracks always tend to gather in the vicinity of major cracks. At comparable strain level, this mechanism would lead to a noticeably larger opening of major cracks. This is in accord with the preceding findings that hybrid fiber ECC exhibits greater permeability than mono PVA fiber composites at large strain level.

Regarding the difference between two hybrid fiber reinforced systems, steel fiber content of 1.0% allows a major inclined shear crack to propagate along the specimen with the cracking plane roughly 30° from the vertical direction, while vertical cracks dominate in specimens with steel fiber content of 0.6%. The underlying mechanism might lie in the fact that larger amount of steel fibers may provide stronger lateral constraint and bridging effect when pull out, altering the failure mode of ECC.

## 4. Summary and Conclusions

In the present research, the stress–strain properties and in-situ gas permeability evolution of PVA-steel hybrid fiber reinforced engineered cementitious composites under both uniaxial and triaxial compression have been fully investigated. Special focus is centered on the impact of additional steel fiber content and confining pressure on the compressive performance and permeability properties. Thus, two additional steel fiber contents were selected while the PVA fiber content is maintained as 1.7% in the experimental program.

The test results indicate that the compressive performance of ECC can be significantly improved by incorporation of additional steel fibers, in terms of strength, strain capacity and toughness. With the increase of steel fiber content, the ascending slope of stress–strain curves increase slightly, while the descending stage obtains significant promotion, thus yielding higher residual stress. Additionally, the compressive properties of ECC is found to be sensitive to the confining pressure, which attains a substantial increment at a low level of confinement. However, the presence of confining pressure tends to affect the enhancement efficiency of additional steel fiber to some extent.

The permeability evolution of strained ECC corresponds well to the variation of radial strains. It experiences a small change below the threshold stress but a rapid increase beyond the peak axial strain. Incorporation of additional steel fibers is found to play a significant role in the permeability properties. The mono PVA fiber composites show a relatively moderate increment in permeability, whereas the hybrid fiber composites appear to be more permeable all along the loading process. It is in accord with the failure patterns that a multitude of closely spaced but disconnected microcracks form in mono PVA fiber composites, whereas a few larger cracks form in hybrid fiber system. In summary it can be concluded that the addition of sufficient amount of micro steel fiber is clearly beneficial for improving the uniaxial compressive behavior of ECC. However, it should also be recognized that the impermeability of cracked ECC under compression would be impaired by additional steel fibers, especially in the post-peak stage of the stress–strain curves. In regard to the ECC components subjected to triaxial compression, the feasibility of additional steel fibers must be reconsidered. Further experimental and theoretical investigations are also required to characterize the triaxial compressive behavior and the in-situ permeability of ECC under various confining pressures, relating to the toughening effect and durability issue of this innovative material in underground or hydraulic structures.

## Figures and Tables

**Table 1:** Physical properties of polyvinyl alcohol (PVA) and steel fibers.

Fiber Type	Density (g/cm^3^)	Tensile Strength (MPa)	Elastic Modulus (GPa)	Diameter (mm)	Length (mm)
PVA fiber	1.2	1620	42.8	0.039	12
Steel fiber	7.8	2750	210	0.200	13

**Table 2:** Volume fraction of fibers and physical parameters of different engineered cementitious composites (ECC) mixtures.

Mix Name	PVA Fiber in Volume (%)	Steel Fiber in Volume (%)	*ρ* (kg/m^3^)	*k* (×10^−17^ m^2^)
Mono	1.7	0.0	1949	1.20
Hy1	1.7	0.6	1952	1.67
Hy2	1.7	1.0	2026	1.57

**Table 3:** Mechanical parameters of ECC mixtures with different steel fiber content under uniaxial and triaxial compression.

Stress State	Mix Name	*f*_c_ (MPa)	*E*_0_ (GPa)	*ε*_0_ (%)	*ε*_02_ (%)	*ν*_0_
Uniaxial compression	Mono	53.3	18.7	0.447	−0.110	0.165
	Hy1	51.7	18.6	0.456	−0.119	0.157
	Hy2	59.4	21.5	0.459	−0.138	0.179
Triaxial compression	Mono	97.5	19.5	0.540	−0.124	0.187
	Hy1	86.2	18.9	0.591	−0.146	0.187
	Hy2	101.7	19.4	0.638	−0.167	0.177
	(2)					
